# Nutrient requirements and feeding management for ostrich during breeding and production: A comprehensive review

**DOI:** 10.1016/j.psj.2025.105629

**Published:** 2025-08-05

**Authors:** Muhammad Zeeshan Akram, Zeshan Ali, Hassan Jalal, Muhammad Awais Sarfraz, Muhammad Umair Asghar, Abdul Rauf, Ludovica Maria Eugenia Mammi, Paolo Pezzi, Melania Giammarco, Isa Fusaro

**Affiliations:** a Nutrition and Animal-Microbiota Ecosystems Laboratory, Department of Biosystems, KU Leuven, Heverlee 3000, Belgium; b Laboratory for Animal Nutrition and Animal Product Quality, Department of Animal Sciences and Aquatic Ecology, Ghent University, Belgium; c Department of Veterinary Medicine, Faculty of Veterinary Medicine, University of Teramo, Teramo 64100, Italy; d Department of Animal Nutrition, University of Veterinary and Animal Sciences, Lahore 54000, Pakistan; e Department of Animal Nutrition and Feed Science, The Faculty of Biology and Animal Science, Wrocław University of Environmental and Life Sciences, 25 Norwida St., 51-630 Wrocław, Poland; f University of Veterinary and Animal Sciences, Jhang Campus, Jhang 35200, Pakistan; g Department of Veterinary Medical Sciences, Alma Mater Studiorum University of Bologna, Bologna, Italy

**Keywords:** Livestock, Breeding, Meat, Production, Nutrition

## Abstract

Ostrich farming has emerged as a significant contributor to the livestock industry, driven by the high value of its meat, hides, feathers, and oil in international markets. With an average egg production of 40 eggs per season and yielding 58.59% carcass on live weight, ostriches offer substantial economic benefits. The nutritional needs of ostriches evolve across different life stages, impacting growth and reproductive performance. As herbivores capable of digesting high-fiber diets, ostriches thrive on roughage and pasture, with a digestive system similar to other poultry. Essential nutrients, including 20-24% crude protein (CP) and 12-19% crude fiber, are critical for optimal development, particularly in early growth, where their feed conversion ratio is 2:1. As ostriches reach sexual maturity, maintaining a diet rich in amino acids, vitamins, and carbohydrates becomes vital for optimizing fertility and reproductive performance. This review provides a detailed overview of ostrich nutrient requirements across different growth stages, focusing on how energy and protein levels affect overall growth and productivity. It also explores the importance of feeding management practices in enhancing ostrich health and performance, offering insights for improving commercial ostrich farming.

## Introduction

Ostriches, one of the largest living birds, are historically significant and hold great potential in modern livestock production. Smith (1995) identified four species of ostrich: North African (Struthio camelus Linnaeus), East African (Struthio molybdophanes Neumann), Somali (Struthio molybdophanes Reichenow), and South African (Struthio australis Gurney). Historically, ostriches have been significant in various cultures. The ancient Egyptians adorned royal headdresses with ostrich feathers, while Greeks and Romans used them to embellish ceremonial gear. Ostrich eggs served as both utility items and decorative objects in Ethiopian Coptic churches (Meawad, 2020). The first documented commercial ostrich farming began in South Africa during the 19th century, with the industry expanding globally to countries like the USA, Australia, and Egypt (Mahrose et al., 2015). As the demand for protein-rich foods increases with the rapidly growing human population (Akram et al., 2019, 2021; Asghar et al., 2021, 2022; Jalal et al., 2024), alternative farming options, like ostrich farming, have emerged as viable solutions. Research shows that ostrich farming, alongside poultry, can meet the high protein demand effectively (Ramos et al., 2013). Despite its economic significance, research into ostrich nutrition lags behind that of more traditional livestock, such as broilers and pigs (Gous and Brand, 2008). Developing a thorough understanding of their nutritional requirements at various growth stages is vital to improving ostrich performance (Bovera et al., 2014). Ostriches are resilient birds native to the African continent, found primarily in the southern Sahara and extending to Cape Town. Six subspecies of ostrich were described by Ullrey and Allen (1996), although one subspecies is now extinct. Today, ostriches can be found across the globe, and commercial farming has adapted them to various climates, including arid regions. Their natural habitat ranges from semiarid deserts to grasslands, making them suitable for farming in tropical regions (Fair et al., 2012). This adaptability, along with their rapid growth rate and high body mass potential, have solidified their place in the commercial farming industry. For example, ostriches can grow to a weight of 80-100 kg within their first year and can reach an adult height of 6-10 feet by 18-24 months (Bunter and Cloete, 2004). Adult males and females can weigh between 100-130 kg and 90-110 kg, respectively, with carcass yields of 57-58% of live weight (Al-Khalifa and Al-Naser, 2014).

Balancing the nutritional status of ostrich chicks is crucial, as they are particularly vulnerable during their first three months. Poor nutrition can significantly affect their survival rate, growth, and production potential (Bovera et al. 2014). Milton et al. (1994) reported that natural forage for ostriches comprises 70% water, 24% crude fiber, 12% CP, and trace amounts of lipids and ash. Fiber provides approximately 76% of the energy required by ostriches, underscoring the importance of high-fiber diets in commercial settings (Swart, 1988). However, under farming conditions, ostriches require a well-balanced diet supplemented to meet their specific nutritional needs (Fair et al., 2012). Feeding costs constitute the largest expense in ostrich production, with protein being the most costly component (Carstens et al., 2014). Major protein sources in ostrich feeding include soybean meal, peanut meal and fishmeal (Mustafa et al., 2024). Research into optimizing feed for these stages remains limited, necessitating more studies to inform feeding practices and improve outcomes (Bovera et al., 2014). The oesophagus of ostriches lacks a distinct crop that is present in broilers and turkeys (Naser et al. 2024). The submucosa of ostriches is well developed compared to other birds (Naser et al. 2024). The caeca and colon of ostriches are more developed than turkeys and chickens. Paired caeca and colon make up to 2/3^rd^ of the digestive tract (Miao et al., 2003). The proventriculus glands are less developed in ostriches as compared to chickens.

Ostriches, as herbivores, have evolved to thrive on high-fiber diets that include roughage and pasture. Their digestive systems are efficient at breaking down fibrous plant matter, much like other poultry. This capacity to utilize inexpensive feed sources makes ostrich farming economically feasible, especially in regions with limited access to high-quality grain feeds. Factors such as feed quality, social grouping, and genetics have a notable impact on chick growth and overall flock productivity. Seasonal variations and management practices also play critical roles in determining the performance of ostrich flocks (Ramedani et al., 2019).

This review aims to provide a detailed analysis of ostrich nutrient requirements at various stages of growth, from hatchlings to mature birds. By reviewing available data on protein, energy, vitamins, and mineral requirements, this article seeks to identify optimal feeding strategies for maximizing growth rates and reproductive performance. Additionally, the role of feed management in improving carcass yield and overall farm profitability will be discussed. This review contributes to the existing body of knowledge by addressing the gaps in ostrich nutrition research and offering insights into best feeding practices for commercial ostrich farming, particularly in tropical and arid regions. This study will compile the recent literature and recommendations for ostrich feeding, direct future researchers to consider the highlighted research gaps, practical and implementable sustainable farming practices for ostrich rearing. It will also be a source of detailed and literature-based guidelines in ostrich nutrition management at the commercial level.

## Meat quality of ostriches

Ostrich meat has gained attention as a healthy alternative to traditional red meats due to its low fat content (0.34% or up to 3g/100 g) and high protein level (16.5% – 22.26% or 22-24g/100 g), outperforming beef in both categories, up to 10-15g/100 g and 19-22g/100 g for fat and protein respectively (Table 1) (Asif Arain et al. 2025, Dalle Zotte et al., 2013; Henry et al., 2024). Ostrich muscles also contain high concentrations of myoglobin (1.14%) as compared to goat (1.07%), contributing to iron storage, with levels ranging from 20-30 micrograms of Fe/g on wet weight basis (Medina et al., 2014). The meat is rich in favorable omega-3 fatty acids with lower n6:n3 (3.02) as compared to poultry (7.67) and beef (8.86) (Asif Arain et al., 2025), which align with dietary recommendations by WHO for prevention of chronic diseases to reduce saturated fat intake in favor of polyunsaturated fatty acids (PUFAs) to lower the risk of coronary heart disease (WHO 2003, Henry et al., 2024; Cooper and Horbañczuk, 2002). Ostrich meat is considered an ideal choice for health-conscious individuals who prefer red meat. The World Health Organization (WHO) recommends a polyunsaturated to saturated fatty acid (P/S) ratio of 0.4–0.5 for a healthy diet (WHO 2003). Ostrich meat meets this criterion, with a P/S ratio of 0.53 (Henry, et al., 2024). Ostrich meat is characterized by a higher polyunsaturated fatty acid (PUFA) content compared to beef, chicken, and turkey, as well as a lower sodium content in ostrich meat (43.5-57.85mg/100 g) as compared to turkey (123mg/100 g), poultry (64-83mg/100 g), and beef (65-89mg/100 g) (USDA Food Composition Database, 2018; Henry et al., 2024; Asif Arain et al., 2025), making it a healthier option for individuals with hypertension (Brassó et al., 2020; Henry et al., 2024). The PUFA ratio in ostrich meat fat has been reported as 27.54 ± 1.01, alongside a notable mineral content, further enhancing its nutritional value (Brassó et al., 2021). Its high natural water-binding capacity and relatively high mean pH of 7.2, which increases post-24 hours of slaughtering, further enhance its suitability for processing (Poławska et al., 2011), reducing the need for artificial moisture retainers like phosphates (Sales, 1998). Additionally, ostrich meat contains higher phosphorus levels of about 213mg/100 g compared to chicken (173mg/100 g), beef (150-171mg/100 g) and goat meat (154mg/100 g) (Asif Arain et al., 2025; Henry, et al., 2024). Its low cholesterol content, accounting for only 1.08% of total meat fat or approximately 65.63 mg per 100 g, combined with its unique taste being more juicy, tender, and having fish aroma, has contributed to its growing popularity among health-conscious consumers (Henry, et al., 2024; Horbañczuk et al., 1998). Interestingly, fluctuations in dietary protein level up to 22%, 24% and 26% or energy level up to 2400kcal/kg or 2600kcal/kg have no significant effect on cholesterol levels in ostrich meat (Nikravesh-Masouleh et al., 2021). Polat et al. (2003) also observed no significant difference in the cholesterol profile of ostriches fed with 23% and 20% crude protein feed. Additionally, ostrich meat is rich in phospholipids, which are known to reduce cholesterol absorption in humans by disrupting micelle formation and downregulating cholesterol transporters (Yang et al., 2022; Jesch et al., 2017). Beyond meat, ostrich offal contains a balanced ratio of micro- and macro-minerals, further enhancing its nutritional profile (Henry, et al., 2024). However, the optimal slaughter age for ostriches, typically suggested between 10 and 14 months, remains a topic of debate as it may influence meat quality (Brassó et al., 2021) but recent study by Bo et al. (2025) suggests the best slaughtering of 9^th^ to 10^th^ month depending on the growth curve. Brassó et al. (2021) also observed a similar meat profile of ostriches of 18 months on slaughtering as compared to that of 10-14 months old. Hence, authors go with the opinion of the best slaughtering age of 10 months. Notably, ostriches exhibit a higher dressing percentage (∼59%) compared to beef (∼55%), mutton (38% to 57% depending on breed), and Emu (∼47%), but less than poultry (∼73%) (Coyne et al., 2019; Simela et al., 2011; Abbas et al., 2018; Horbańczuk et al., 2016; Lilic et al., 2021).

**Table 1:** Nutritional profile of ostrich meat.

Constituent	Proportion	References
Dry matter (g/100gm)	22.25, 23.3 – 25.5	(Henry et al. 2024; Brassó et al. 2021; Majewska et al., 2009)
Fat (g/100gm)	0.9 – 1.34	(Majewska et al., 2009)
	1.28, 1.29	(Henry et al. 2024; Horbanczuk and Cooper, 2001) (Brassó et al., 2021)
Ash (g/100gm)	0.97, 1.07 – 1.17	(Henry et al. 2024; Majewska et al., 2009)
Protein (g/100gm)	18.40 – 21.7	(Henry et al. 2024; [Bibr bib0042][Bibr bib0119])
Cholesterol (mg/100 g) (% of total lipids)	57	Horbanczuk and Cooper, (2001)
	57 1.08	(Sales, 1996a) (Henry et al., 2024)
	65 – 68	(Horbanczuk et al., 1998)
	83	(Cooper, 1999)
Fatty acids (%)	30 36.11–56.88	(Horbanczuk et al., 1998) (Henry et al., 2024)
PUFA (% of total lipids)1	35.1 19.27 27.54	(Sales, 1998) (Henry et al., 2024) (Brassó et al., 2021)
Sodium (mg/100 g)	43 57.85	(Sales, 1996b) (Henry et al. 2024)
Iron (mg/100 g)	2.3 8.43	(Sales, 1996b) (Henry et al., 2024)
Phosphorous (mg/100 g)	185.3–329.8	(Henry et al., 2024) (Poławska et al., 2014)

1 = Polyunsaturated fatty acid

## Health challenges during production cycle

Husbandry challenges, such as the failure of chicks to absorb the yolk sac in their early days may hinder growth and development. High ambient temperatures, energy-rich diets, and lack of exercise are contributing factors to this phenomenon (Shanawany, 1996). Average temperature is suggested to be kept 35^◦^C during the first week and then decreasing by 3^◦^C each week for rearing and maintenance at 20 ^◦^C for egg production (Previti et al., 2025; Schou et al., 2022). Suboptimal nutrition, combined with overcrowding, pecking behaviour, inadequate ventilation, excessive heat, and poor husbandry practices, can result in high chick mortality, nutritional stress, and increased susceptibility to diseases. Gastric impaction, often linked to poor management, is a significant contributing factor (Okasha et al., 2019; Irfan et al., 2020; Aryal and Khanal, 2022; Farooq, 2024). Gastric impaction during the first six months of growth has been reported in 30–46% of ostriches (Irfan et al., 2020), with contributing factors including ingestion of foreign bodies, fungal infections, and poor feeding strategies (Irfan et al., 2020). Additionally, Brand et al. (2020a) reported a mortality rate of 11% due to gastric impaction in ostriches aged 2–11 months. Early growth rates are crucial for ensuring healthy ostriches reach slaughter weight (Miah et al., 2020). Bacterial pathogens such as *Escherichia coli, Campylobacter jejuni, Pseudomonas aeruginosa, Salmonella spp*., and *Clostridium* spp. are commonly implicated in ostrich enteritis (Videvall et al., 2019, 2020; Li et al., 2024). Multidrug-resistant strains of *E. coli* have been reported in Iran and Romania against enrofloxacin, doxycycline, and amoxicillin, highlighting a significant health challenge (Iorgoni et al., 2025; Edalatian Dovvom et al., 2024). Asadi et al. (2024) also reported 43% prevalence of *E. coli* in ostrich feces in Iran and resistance against tetracycline and clindamycin. Torkan et al. (2024) reported the presence of resistant *E. fergusonii* up to 9.4% in suspected samples with resistance against amoxicillin, trimethoprim-sulfamethoxazole, and doxycycline. Niculae et al. (2024) also reported 97.6% asymptomatic presence of *E. coli* strains in Ostrich in Romania with resistance against streptomycin, tetracycline, colistin, doxycycline, and ampicillin. Asmaa et al. (2016) reported the prevalence of *E.* coli and *Salmonella* species up to 58-62% and 20-24%, respectively, in Egypt. Azam et al. (2023) reported the prevalence of *E. coli* and *Salmonella* species 40-53% and 46-60%, respectively, in Bangladesh. This study reported resistance against piperacillin, bacitracin, tetracycline, cloxacillin, novobiocin, and cefixime in *E. coli* and against tetracycline, piperacillin, bacitracin, chloramphenicol, and methicillin in *Salmonella.* Hence, the ostrich population is prone to multidrug-resistant pathogens, especially *E. coli* and *Salmonella* species. Microbial contamination during the life cycle has been implicated in an embryonic death rate of 49.8% (Aryal and Khanal, 2022). Viral diseases such as Newcastle Disease are particularly critical (Abbas and Abbas, 2018), with mortality rates reaching 80–85% in ostriches aged 3–6 months in various regions (Siddique et al., 2020). Seasonal variations also influence early growth rates (Brassó et al., 2022). Brassó et al. (2022) elaborated on the significant impact of season on ostrich development and included it in defining relative risk. This study showed that relative risk was more in autumn and thus, hatching rate was minimum. Season impacts drinking and foraging behaviour and alleviates them depending on regional seasonal patterns (Dikmen, 2024). Less eating, drinking, and foraging behaviour is observed at noon; when there is less humidity and more temperature (Dikmen, 2024) and the opposite is the case at late and early in the day; when humidity and temperature are moderate. The summer season also offers ostriches (Dikmen, 2024) prolonged resting and sleeping time. Leg deformities, often resulting from poor nutrition, are another common issue leading to early chick mortality (Brassó et al., 2020; Brassó et al., 2022). Elobeid et al. (2022) found that growing ostriches spent only 20% of daylight hours resting, suggesting that reduced resting time, indicative of higher activity levels, correlates with a healthy musculoskeletal system, an essential factor in successful ostrich farming. Tendon deformities have been associated with 13% lameness in ostrich chicks by 12 weeks of age, though without any reported mortality (Miah, et al., 2020). The duration of active hours varies with seasons and may serve as a valuable parameter for assessing health status in ostrich management (Dikmen, 2024). Despite the economic importance of ostrich farming, research on their nutritional needs lags behind other livestock species like broilers and pigs (Gous and Brand, 2008). The literature has little information about the impact of specific vitamins or minerals on ostrich egg production, egg quality, hatchability, and daily growth. A wide range of micro pathogens needs more discoveries for AMR patterns, their impact on growth, and pathogenicity, Information about accurate energy and protein levels at different life stages remained limited and feeding requirement for meat and egg production in ostriches, major hurdles and compilation of recent management issues and solutions remained almost unaddressed in recent years. Understanding the nutritional requirements of ostriches throughout different growth stages remains essential for optimizing their development and production efficiency (Bovera et al., 2014). Miah et al. (2020) reported a 100% survival rate in ostrich chicks over 12 weeks, achieving a live weight of over 33 kg when fed a poultry starter diet containing 19% CP and 3000 kcal/kg metabolizable energy (ME), supplemented with 3% dicalcium phosphate.

## Digestive system of ostrich

Ostriches have special digestive system to process fibrous material, due to microbial fermentation colon and cecum. Although ostriches lack a true stomach but rely on gizzard to grind feed efficiently. Diet of ostriches primarily consists of grasses, small invertebrates, and seeds making digestion highly dependent on gut microflora and feed quality (Abbas et al., 2017; Abbas et al., 2018a). Variations in the proportion of soluble and insoluble fiber in the diet have been shown to have no significant impact on its digestion or performance (Mirbehbahani et al., 2020; Abd El-Wahab et al., 2021). Smith (1995) demonstrated the ostrich's ability to effectively utilize roughage, and subsequent studies by Cilliers, (1998) and Brand and Olivier, (2011) further highlighted their proficiency in digesting cellulose and fermentable substrates from fibrous feed. This capacity allows ostriches to thrive in semi-arid environments, such as short grass plains and deserts, where they selectively consume plant material with the aid of their sharp beaks, excellent eyesight, and long necks, which facilitate ground-level foraging (Milton et al., 1994). Ostriches exhibit a diverse gut microbiota, with variations observed across different regions of the gastrointestinal tract (Heitmann, 2020). Similar findings were reported by Videvall et al. (2020) who identified differences in the microbiota of the ileum and cecum between control and diseased groups in early-growing ostriches. The ostrich diet predominantly consists of various plants and succulents, although juveniles may also consume insects for additional protein (Milton et al., 1994). Literature for diversity of favourite insects is limited but Microhodotermes, the termites are reported to be selected by juvenile ostriches (Milton et al., 1994), Adult ostriches are mainly herbivorous, supplementing their diet with grit and small stones to aid in grinding food, similar to other bird species (Abd El-Wahab et al. 2021). Toxic plants and indigestible fibrous grasses are typically avoided (Williams et al., 1993). Their digestive system includes a relatively short small intestine and a well-developed large intestine, featuring an extended cecum where fermentation of fibrous food occurs, aiding in fluid absorption and nutrient extraction (Shanawany, 1996). Jimenez-Moreno et al. (2013) reported that increasing sugar beet pulp from 2.5 to 7.5% in broilers’ diet resulted in reduced weight gain. Also, 3 to 9% increase in fiber content caused a decrease in turkeys’ growth performance (Sklan et al., 2003). However, ostriches’ colon microbiota is capable of digesting 38% cellulose and 68% hemicellulose (Swart et al., 1993b). An association has been observed between the development of digestive organs and diet ingested by ostriches (Abd El-Wahab, et al., 2021).

## Ostrich feeding management

Ostriches typically consume a variety of plant materials, including grass, fruits, flowers, leaves, and grains. The selection of feed and sources of CP and ME is crucial for the proper development of vital organs, such as the liver and thyroid (Brand et al., 2020a). For commercial ostrich farming, poultry feed is commonly used, and during the breeding period, the diet is adjusted with an additional 2.8% calcium to support egg production (Gandini, 1986). For non-egg-producing ostriches, calcium levels of 1.0%–1.5% and phosphorus levels of 0.8%–1.0% are considered sufficient for optimal health (Brand et al., 2020c). Calcium and phosphorus deficiencies are known to cause leg abnormalities in ostrich chicks, especially from 2 to 26 weeks of age, leading to issues such as tibiotarsal rotation (Bezuidenhout et al., 1994). Notably, young chicks show a preference for feeding directly from the floor rather than trays, with studies revealing that 50.7% of chicks preferred pellets scattered on the floor (Bubier et al., 1996; Paxton et al., 1997). Proper management during the early stages of life is crucial for chick survival and growth. For the first three weeks, authors suggest that chick pens and feeding systems should remain constant, and pens should be connected to brooder buildings to protect the chicks from adverse weather conditions. By six months of age, ostriches typically spend about 80% of their time in outdoor pens, where their activities are dominated by walking, sitting, and feeding (61.5%, 20.4%, and 6.6%, respectively) (Degen et al., 1989). Tomuta et al. (2021) observed walking behavior as 20% of routine activities.

The growth of ostrich chicks follows a distinctive pattern. At birth, chicks weigh around 4080 g, with an initial weight loss of 7.6 g/day in the first week, followed by gains of 43.4 g/day in the second week, 114 g/day in the third week, and 145 g/day from 21 to 35 days (Degen et al., 1991; Miao et al., 2003). Birth weight in ostriches can vary, as Brand et al. (2020b) reported a birth weight of 0.89 kg for ostrich chicks. The growth potential of ostriches is significant, with males reaching up to 115 kg and females up to 114 kg at around 181 and 199 days, respectively at 23-25 ^◦^C temperature, lucerne pastures, semi controlled environment widening with growth period, ad libitum food and water, with apparent metabolizable energy and protein starting from 12.5 MJ/kg and 230g/kg and finishing on 7 MJ/kg and 100g/kg, respectively (Cilliers et al., 1995). Feed conversion ratios also vary with age. In the early stages, a feed conversion ratio (FCR) of 2:1 is achievable, while this ratio decreases to 5:1 when birds reach around 70 kg (Farrell, 1997). By 10 to 14 months, FCR may decrease further to 10:1. The best growth results in ostriches are achieved with a diet containing 14% protein and 35% lucerne, providing 10.8 MJ of ME per kg (Swart and Kemm, 1985). Nikravesh-Masouleh et al. (2021) reported the best FCR at a ME level of 2600 kcal/kg, optimal body weight at a lower ME of 2400 kcal/kg, and reduced feed intake at an elevated CP level of 26%. However, Mahrose et al. (2019) found no significant effect of low CP (>180 g/kg) on feed intake, FCR, or protein efficiency when compared across different stocking densities in ostriches. Hence, higher protein content (more than 14%) is unnecessary for birds in the 60 to 110 kg live weight range, as it does not yield significant improvements in growth. Feed intake in ostriches can be influenced by seasonal variations, though this aspect is often underreported or neglected in studies. Mutiga et al. (2016) observed higher feed intake in ostriches during the dry season, while Dikmen, (2024) found no seasonal effect on digestion and related activities. Nikravesh-Masouleh, et al. (2018) reported increased weight gain in ostriches fed a diet with comparatively low ME (10.1 MJ/kg). Additionally, ostriches lack sufficient trypsin enzyme secretion immediately after birth, which may contribute to inefficient protein utilization during the early stages of growth (Mahrose, et al., 2019). Proper feeding practices, such as distributing feed to ensure that chicks have equal access, are essential for optimal growth (Morris et al., 1995; Bubier et al., 1996; Lambrechts et al., 1998). It is recommended to use one feed bowl for every five chicks for optimal feeding (Hayter, 1998).

Maximum feed intake in ostriches was observed at different growth stages: from 0-1 month (pre-starter) with 14.5 MJ/kg ME and 190 g/kg CP, from 1-3 months (starter) with 10.5 MJ/kg ME and 160 g/kg CP, from 3-6 months (grower) with 8.5 MJ/kg ME and 195 g/kg CP, and from 6-9 months (finisher) with 7.5 MJ/kg ME and 80 g/kg CP (Brand et al., 2020c). The authors found no significant effect of feed intake on weight gain, suggesting that variations in CP and ME levels above optimal thresholds are unlikely to alter live weight in ostriches, although increased energy intake may promote fat deposition (Brand et al., 2019; Brand, et al., 2020c). However, the study focused solely on empty body protein weight (EBPW). In another study, ostriches fed a pre-starter diet with 20% CP and 14.0 MJ/kg ME from day 0 to 76 gained an average weight of 4.53 kg, starting from 0.89 kg on day 0. This study also compared two different protein sources, soybean meal and canola meal, for ostrich growth (Brand, et al., 2020a). Pre-starter feed, which contains 20.5-22% protein, has been shown to improve the growth performance of ostrich chicks from day-old to 6-8 weeks, with feed intake ranging from 22-25 kg per period. Starter feed containing 18-20% protein is recommended for older chicks, with feed intake increasing to 55-60 kg per period (Cilliers, 1988). By the age of 14 months, ostriches can yield approximately 35 kg of meat, with a lean meat production rate of 62.5%, higher than that of broilers and beef (65% and 64%, respectively) (Morris et al., 1995). Once an ostrich reaches a live weight of 85 kg, it is ready for slaughter (Jones et al., 1995), and the process of slaughtering ostriches is well established in commercial operations, such as the abattoir in South Africa, following strict standards (Hoffman, 2012).

### Starter

Immediately after hatching, ostrich chicks typically lose up to 20% of their weight within the first 5 to 7 days, with this decline being more severe and prolonged in sick birds (Deeming et al., 1993). Once this phase passes, chicks begin to steadily gain weight at an increasingly faster rate. By the age of three months, healthy chicks are expected to weigh between 35 and 40 kg, eventually reaching an adult weight of around 100 kg by 12 months of age (Degen et al., 1991). Brand, et al. (2020a) reported a weight of 100 kg in ostriches within 337 days by using a mixture of soybean meal, lucerne meal, and canola meal as the protein sources in their feeding regimen. Newly hatched chicks primarily rely on the yolk sac for nourishment during the first 24 to 72 hours, with the sac remaining for 10 to 14 days post-hatching (Deeming et al., 1993). They also observed that chicks initially lose weight for the first five days after hatching, then begin to gain weight consistently, reaching at least 4 kg by five weeks of age. Balanced nutrition and careful management during this early stage, up to three months of age, are critical to prevent issues such as growth retardation and skeletal disorders (Shanawany, 1996; Cooper, 2000).

Interestingly, wild ostrich chicks ingest their parents' manure as their first food, a behavior that introduces beneficial bacteria needed to digest plant fiber (Cooper et al., 2004). Diversity in gut microbiota plays a crucial role in preventing early chick mortality (Videvall, et al., 2020). Additionally, nocturnal coprophagy was observed in ostriches during the growing period (i.e., >2 months of age) in a recent study (Elobeid, et al., 2022). Schmitt, (1997) recommended offering a pre-starter ration with high energy content (55% grain, 22% protein, and 20% roughage) from day one, which resulted in chicks gaining an additional 18 kg by eight weeks of age. Authors also emphasized the importance of essential amino acids, including arginine, lysine, cysteine, and methionine. (Brand, et al., 2022) emphasized the inclusion of salts in the feed for early growth, finding that a high salt content (14 g/kg) resulted in increased daily weight gain in ostriches up to one month of age. In the first 7–10 days after hatching, chicks rely more on water than food, due to their dependence on the remnants of the yolk sac for nourishment (Shanawany, 1996). Water intake in ostrich chicks is influenced by factors such as age, weather, and feed ration, as reported by (Cooper, 2007). Chicks are notably attracted to green plants during feeding (Bubier et al., 1996), and they engage in coprophagy, a natural behavior where they eat feces, which introduces beneficial microorganisms to their digestive system. Previous studies demonstrated the negative impact of a high-fiber diet on young ostrich chicks, reporting that fiber digestibility was as low as 6.5% at three weeks of age due to intestinal obstruction (Angel, 1993; Cooper and Palmer, 1994). Scheideler, (1994) suggested that starter rations should not contain more than 0.8% fiber until chicks reach three months of age to avoid digestive issues. Furthermore, a study by (Jacomina Aletta, 2016) highlighted that the digestibility of fiber content is influenced by the neutral detergent fiber (NDF) content in feed. Supporting this, several studies suggest that incorporating forages into ostrich feed prolongs the exposure of feed contents in the stomach, thereby promoting optimal digestion (Abd El-Wahab, et al., 2021; Afshin, et al., 2024; Jacomina Aletta, 2016).

### Grower

At 16 weeks of age, when ostriches reach a body weight of around 45 kg, a grower ration is introduced that includes fiber from high-quality forages such as chopped lucerne. Ostriches possess a more efficient digestive system compared to chickens, allowing them to digest a wider range of foodstuffs (Cooper et al., 2005). To support maximum body growth, the grower ration should provide approximately 40% energy and 16% protein. (Salih et al., 1998) conducted a trial examining the effects of grower diets on dry matter intake (DMI) and fat absorption in ostriches at 13 weeks of age. They offered diets containing 17.1% protein with 0.97% lysine and ME contents of 14.0, 11.5, and 9.0 MJ/kg up to 20 weeks of age. The study observed that DMI was higher in birds fed medium-energy diets (1916 g/day) compared to those on low-energy diets (1510 g/day). (Elobeid, et al., 2022) utilized a grower feed with 16.34% CP and 10.11 MJ ME/kg for ostriches older than two months. However, the study did not evaluate the relationship between feed composition, health, or weight gain. Furthermore, fat absorption, which was initially 44.1% at three weeks of age, increased significantly to 92.9% by 30 weeks of age. (Cooper et al., 2005) emphasized the importance of meeting the specific nutritional needs of growing ostrich chicks, as their growth rate is fastest during this period. According to their findings, the energy requirement for growth peaked at 10.689 MJ/day for ostriches at 180 days of age. The total energy requirement continues to increase linearly, reaching a peak of 19.36 MJ/day at around 300 days. At 10 months of age, 70% of roughage is incorporated into the finisher ration, which also contains 25% grain and 14% protein. (Schmitt, 1997) reported that the finisher diet used before slaughter predominantly consisted of roughage (around 90%), while the maintenance ration offered during the nonbreeding season contained 10-12% protein for birds weighing between 10-120 kg. This balance supports proper growth, fattening, and health during the finishing and nonbreeding phases of ostrich production.

## Ostrich breeding and production

Sexual maturity in ostrich females typically occurs between 18 and 24 months at farm conditions (Abbas et al., 2018; Prokopenko et al., 2021; Tomuta et al., 2021) with egg production commencing around 733 ± 83 days of age (Cloete et al., 2014; Cooper et al., 2005). Reproductive life in ostriches may remain optimal beyond 8-9 years, as (Prokopenko et al., 2021) compared egg-laying capacities of Black African ostriches under semi intensive systems (open air cages) at this age with sex ratio of 1:3 and 1:2 and found them to be similar to those of younger ostriches (4-5 years) and this capacity showed positive correlation between duration of egg-laying process and total egg-laying performance. This study did not reveal the nutritional profile of given feed to laying ostriches. However, eggs from the first production cycle are not typically viable for hatching (Tomuta, et al., 2021). Additionally, Prokopenko et al. 2021 compared ostriches aged 4-5 years, suggesting that the first production cycle could have been excluded from their analysis. Female ostriches can produce eggs every two days, though individual production rates can vary significantly (Elsayed, 2009). Ostriches have polygamous (a dominant male mates with multiple females) mating system. The reproductive success depends on multi-factors i.e. genetics, nutrition, and environmental conditions (Abbas et al., 2017; Abbas et al., 2018a). Ipek and Șahan (2006) reported an average of 40-60 eggs during the mature breeding season (from the first to subsequent years), while (Abbas et al., 2018) estimated an average of 30 eggs per annum. Prokopenko et al. (2021) observed an average of 30 eggs over a full breeding season, comparing two age groups of female ostriches, and found higher egg weights in older females. Average egg weights were reported as 1300-1400 g by Prokopenko et al. (2021) and 1200-1800 g by Abbas et al. (2018). However, laying capacity may vary depending on strain (genetic potential) (Brand and Olivier, 2011). Mating frequency, which is also influenced by seasonal factors such as expression of more copulation at noon in autumn and at afternoon in summer season (Dikmen, 2024), secretion of gonadotrophin releasing hormone is affected by photoperiod (Johnson, 2015) plays a key role in reproductive success (Dikmen, 2024). Interestingly, females can remain fertile for up to 40 years (Cooper, 2000) or 30 years (Abbas et al. 2018), making them a valuable long-term investment for breeders. Both Cooper (2000) and Abbas (2018) did not explain genetic reasons or environmental factors for this difference and reported fertile life separately. However, nutritional depletion, such as daily energy intake less than 20.6 MJ ME/d and protein content less than 64g/kg (Olivier et al., 2024), lack of vitamins premix in feed (Tesselaar et al., 2015) in females may induce the laying of poor-quality eggs and compromised hatchability including dead in shell (DIG) eggs. Brand et al. (2014) opposed these findings and revealed no effect of dietary supplementation of mineral premix and trace elements on eggs’ production or hatchability in ostriches. The natural incubation period for ostrich eggs is typically 41-43 days and both parents (male and female) participate in incubation and chick care (Tomuta et al. 2021; Abbas et al. 2018). During this time, the activity of parent birds becomes more pronounced and protective. Female ostriches have an extended breeding season, lasting more than 8 months usually starting from spring to the end of summer months and highly dependent on durability of sunlight (Prokopenko et al. 2021), which emphasizes the importance of maintaining ideal nutrition (Cooper et al., 2005). Poor feeding practices have been identified as a major factor contributing to low breeding success in the past (Brand et al., 2000). Optimal breeder performance has been observed with diets consisting of 35% dried lucerne and 14% protein (Swart and Kemm, 1985). Olivier et al. (2009) and Brand et al. (2015) reported that feeding laying ostriches more than 25.5 MJ ME and 75-140g/kg CP per bird does not significantly impact production. Olivier et al. (2024) suggested a CP level of 160g/kg per laying bird for production maintenance. Furthermore, the optimum ME value for laying ostriches is proposed to be 22 MJ per bird (Brand and Olivier, 2011). Beyond these values, further additions in ME and CP showed no benefit in the birds’ performance in the corresponding studies. Regardless of the ME and CP levels, ostrich breeding is considered independent of seasonal variations in feed requirements, as the same amount of energy and nutrients is needed year-round (Olivier et al., 2009; Olivier et al., 2024). The calculated values for energy requirements by Olivier et al. (2024) are similar to the provided by Brand et al. (2003) for breeding females. Calculated values for total protein required daily for breeding ostriches by Olivier et al. (2024) remained well aligned with Du Preez (1991). Studies calculating and discussing energy and protein requirements for breeding as well as resting seasons remained limited in literature. However, Du Preez et al. (1991) also tabulated the energy requirements for breeding ostriches at different weights, and there were few differences in the values. In fact, 1.5 MJ was added for 10 kg difference in birds’ weights. Also, Du Preez et al. (1991) showed variation in energy requirements depending on the egg shell weight in breeding ostriches, and 0.4 kg increase in egg shell’s weight increased energy requirement by 2 MJ. Additionally, egg composition has been shown to be unaffected by bird age and season (Olivier et al., 2024).

In the wild, ostriches generally take 3 or more years to become sexually mature and ready for breeding, while proper nutrition and commercially available feed can induce earlier maturity, around 2 years of age (Brand and Olivier, 2011). Once breeding begins, hens lay approximately 140-505 eggs with an average laying interval of 5-7 days (Brand et al., 2010). Gandini et al. (1986) provided growing ostriches with 14g/kg of feed and still the birds suffered from leg deformities during development. Since egg production requires even more calcium supply, ostrich hens should be provided with dietary calcium of around 16 g/kg, with ad libitum access to calcium sources such as granulated calcium carbonate or oyster shells (Ullrey and Allen, 1996; Hallam et al., 1992). Gandini et al. (1986) added 25g/kg calcium in ostrich diet, however, excessive calcium intake may reduce the absorption and utilization of zinc and interfere with the intake of other essential minerals. Therefore, for laying hens, the dietary level of calcium (dry matter basis) is recommended to be 1.65% (Aganga et al., 2003). Aganga’s recommendation aligns with Ullrey and Allen. (Shanawany 1996) found that as ostriches reach maturity, calcium and phosphorus levels in their diets must be increased to at least 4.0 and 4.2 g/kg, respectively. Although required calcium levels for growing and laying ostriches are found in the literature, the specific effect of deficient calcium on egg shell quality or egg quality remains unhighlighted in the literature for ostriches as compared to the literature present for other poultry birds. However, calcium metabolism in egg content may be influenced by the presence of vitamin D (Mitchell and Edwards, 1996). Hence, level of calcium deposition in egg content and egg shell may influence hatchability. Brand and Steyn (2002) also highlighted that similar feed quantities were required during the breeding season, regardless of whether males and females were housed together for mating purposes.

Nutritional needs during the breeding period are crucial for egg production and hatchability. Lambrechts et al. (1998) found that follicular growth in birds generally occurs between 7 and 8 days, but in ostriches, follicular growth can be extended up to 18 days when proper nutrition is provided during breeding. Polat et al. (2003) discovered that excessively high levels of CP in the diet negatively affected the number of eggs and hatchability rates. Niemann (2018) also reported that CP level up to 28% leads to health and cost issues. Brand et al. (2012) found no significant impact of CP level on laying performance. Thus, they concluded that the CP content in ostrich breeder diets should not exceed 20% to optimize reproductive performance. CP levels exceeding 20% in ostrich breeder diets may impair reproductive performance, likely due to increased nitrogenous waste and metabolic burden, which can negatively affect fertility and egg production. Mahrose et al. (2015) also found decreased tibiotarsus girth with CP level 24% and optimum results were achieved in the group fed with CP level 18% in growing ostriches. Mahrose’s recommendation aligns with the findings of previous study (Gandini et al., 1986), which favored the best FCR at 18% CP level.

### Influence of protein and energy on ostrich breeding

Egg production in ostriches is influenced by several factors, including feeding, genetics, climatic conditions, and seasonal changes. Effects of protein and energy levels on reproductive performance, egg production and characteristics is presented in Table 2. Horbanczuk (2002) reported that, on average, ostriches in South Africa produce around 60 eggs per season, while in European countries, the number ranges from 35 to 50 eggs per female per season. Preez (1991) conducted a study to evaluate egg production, female body weight gain, and the rate of lying down (no activity), also exploring the protein and amino acid requirements of breeding ostriches. Similarly, in an experiment where soy oil cake was replaced with cottonseed oil cake, it did not affect egg production (Brand et al., 2015) . Studies on male ostriches have shown similar results. For example, Cecil (1981) tested various protein levels (8%, 11%, 13%, and 17%) in male ostriches and found no influence on semen quality, fertility, or hatchability. High levels of feeding in ostriches can lead to obesity, which decreases libido (Aganga et al., 2003). Trials based on low-protein diets also demonstrated no effect on infertility or reproductive performance in male or female ostriches during the breeding season (Brown et al., 2002). However, Morris and Gous, (1988) observed that low-protein diets reduced egg weight and the proportion of eggs laid. Brand et al. (2015) recommended that breeding ostriches should receive 200 g of protein daily, including 8.8 g of lysine, for maintenance and egg production. Excessive protein intake could affect body growth and egg production, with excess protein broken down and excreted in urine (Dryden, 2008).

**Table 2:** Effects of energy and protein on ostrich breeding.

Performance objectives	Protein					References
	Protein Levels (g/kg)	Optimum	Age	Sex	Effects	
Egg production	75,91,108,123,140	91.0	2-12 year	Female	+23.5	Brand *et al.* (2015)
Un fertilized eggs (/bird)	75,91,108,123,140	91.0	2-12 year	Female	+13.5	Brand *et al.* (2015)
Egg weight, (g)	13.5,15.0,16.5	13.5	ND^1^	Straight run	+0.75	Brand *et al.* (2003)
Live chicks hatched, (%)	13.5,15.0,16.5	15.0	ND	Straight run	+20.5	Brand *et al.* (2003)
Yolk weight, (g)	105,120,135	105	ND	Straight run	+3.9	Brand *et al.* (2003)
Albumin weight, (g)	105,120,135	135	ND	Straight run	+10.9	Brand *et al.* (2003)
Egg production	10.5,12.0,13.5	12.0	ND	Female	+19.6	Brand *et al.* (2002)
Egg weight, (g)	10.5,12.0,13.5	10.5	ND	Straight run	+2.5	Brand *et al.* (2002)
**Performance objectives**	**Energy**					**References**
	**Energy Levels (MJ ME/kg)**	**Optimum**	**Age**	**Sex**	**Effects**	
Egg production^2^	8.0,8.7,9.4,10.1,10.8,11.5	10.1	2-10 year	Female	+30.6	(Brand et al., 2010)
Egg weight (g)	8.0,8.7,9.4,10.1,10.8,11.5	8.0	2-10 year	Female	+4.4	(Brand et al., 2010)
Infertile eggs^3^	7.5,8.0,8.5,9.0,9.5,10.0	8.5	2-12 year	Female	+39.6	(Olivier et al., 2009)
Hatchling mass (g)	8.5 9.5 10.5	10.5	ND	Straight run	+1.8	(Brand et al., 2003)
Yolk weight (g)	7.5,8.5,9.5	9.5	ND	Straight run	+3.9	(Brand et al., 2003)
Albumin weight (g)	7.5,8.5,9.5	7.5	ND	Straight run	+10.9	(Brand et al., 2003)
Live chicks hatched	7.5,8.5,9.5	8.5	ND	Straight run	+1.9	(Brand et al., 2002)

1 = not determined; 2 = eggs/female/season; 3 = eggs/female/season

The impact of energy intake on body weight gain during the breeding season has also been extensively studied. Brand et al. (2012) found that when ostriches were offered ad libitum feeding with energy levels of 8 and 11.5 MJ ME/kg and a consistent protein level of 120 g/kg, female ostriches gained 5.3 kg with the lower energy level and 15.6 kg with the higher energy level by the end of the breeding season. Brand et al. (2015) further studied the seasonal effects of energy levels on body weight during breeding periods. Provision of 2.5 kg of feed with 18.8 MJ ME per day resulted in a weight loss of 24 kg by the end of the season. In a another study, a daily intake of 23.8 MJ of ME energy was provided to maintain body weight across breeding seasons; while this level was sufficient during the first season, it led to a 12 kg body weight loss in the second season, indicating inadequate energy provision over time. Meeting the energy demands of breeding ostriches is critical to prevent weight loss and ensure optimal performance. In this context, Bels (2006) also demonstrated that female ostriches require approximately 22 MJ ME/day to maintain energy balance during the breeding season. Brand et al. (2003) also noted that poor body growth and reduced egg production occurred in female ostriches fed lower energy (8.5 MJ ME/kg) and protein (105 g/kg) diets. These findings highlight the critical role of balancing protein and energy intake for optimal egg production and body weight maintenance in breeding ostriches. Excessive or insufficient levels of protein and energy can lead to undesirable weight changes and reduced reproductive performance, particularly during the breeding season.

### Influence of energy on body characteristics and growth performance

Ostriches have the capacity to consume more energy and dry matter from feed ingredients compared to adult roosters (Olivier et al., 2024). However, determining the appropriate energy values to maintain a balanced ration for ostriches is challenging, as there is limited research on the impact of energy on digestibility and growth performance. Effects of different energy levels on growth performance of ostriches are presented in Table 3. Maize meal is considered an optimal source of concentrated energy for ostriches. Growth performance in ostriches is influenced by energy concentration and is largely dependent on their age, live body weight, and the phase of growth. Nikravesh-Masouleh et al. (2018) evaluated the effect of dietary energy levels on ostrich growth performance. The authors reported that offering ostriches a diet with 2600Kcal/kg of energy and 24% protein led to low body weight gain in growing ostriches (2-9 weeks). In contrast, Tasirnafas et al. (2015) compared 2500kcal/kg and 2700kcal/kg of energy and found no results on weight gain or feed conversion. Niemann (2018) compiled total metabolizable energy requirements in ostriches from 0-12 months nominated as pre starter (0-2 months), starter (2-4.5 months), grower (4.5-6.5 months), finisher (6.5-10.5 months) and maintenance (10.5-12 months) as 14.5, 13.5, 11.5, 9.5 and 8.5MJ/kg, respectively (Brand and Gous 2006; Brand and Olivier 2011; Brand 2016). Brand et al. (2020c) attributed the increased EBPW achieved with low-energy feed to decreased feed intake at higher energy levels. In contrast, Brand et al. (2019) observed greater feed intake with elevated CP levels (12-22.8%) across different stages, including starter, pre-starter, grower, and finisher. These results showed similar pattern across all age groups. Similarly, Alagawany et al. (2022) reported increased feed intake in domesticated geese with elevated CP (18%) and ME up to 3000 kcal/kg. This supports Ferguson's hypothesis, which suggests that birds continue feeding until they are satisfied with the limiting factor in the feed (Ferguson, 2006). It means if we limit energy or protein in feed, ostriches may keep feeding until their limiting ingredient’s (energy/protein) amount is sufficiently ingested. Fiber also plays a crucial role in ostrich nutrition, with a considerable portion of energy being derived from volatile fatty acids produced through the fermentative digestion of fiber (Swart et al., 1993a; Bovera et al., 2011).

**Table 3:** Effect of energy on body characteristics and growth performance.

Performance objectives	Energy level tested^1^	Optimum	Age	Sex	Effects	R References
Abdomen circumference	2400, 2600	2400	2^nd^ week	Straight Run	+5.7	(Nikravesh-Masouleh et al., 2018)
Breast circumference	2400, 2600	2400	2^nd^ week	Straight Run	+14.6	(Nikravesh-Masouleh et al., 2018)
Feed conversion ratio	10.5, 11.5,12.5	11.5	1-8 months	Straight Run	-14.1	(Brand et al., 2014)
Carcass weight (kg)	12.5, 13.5, 14.5	13.5	1-8 months	Straight Run	+14.8	(Brand et al., 2014)
Feed intake	10.5, 11.5,12.5	11.5	Adult	Straight Run	+10.4	(Brand et al., 2014)
Body weight gain	8.5, 9.5, 10.5	10.5	Adult	Male	+13	(Brand et al., 2002)
Abdominal girth (cm)	8.5, 9.5, 10.5	10.5	Adult	Male	+08	(Brand et al., 2002)

1 = MJ ME/kg.

Energy requirements for ostriches vary between captive and wild birds. Captive ostriches require 12.7–15.0 MJ/day, while wild ostriches need 15.4–19.0 MJ/day (Milton et al., 1994). Tasirnafas et al. (2015) conducted a trial to evaluate the effect of energy levels and vegetable waste on ostrich growth performance. Their results were best with 2500kcal/kg and 10% vegetable waste addition. Brand et al. (2018) examined how different energy levels affected the production parameters of ostriches during the pre-starter and starter phases. They offered five diets with varying energy levels—ranging from 13.5 to 15.5 MJ/kg during the pre-starter phase (0–83 days) and 12.5 to 14.5 MJ/kg during the starter phase (84–149 days). The highest weight gains (22.3 kg and 51.5 kg) were observed in birds fed diets containing 14.5 MJ/kg during the prestarter phase and 13.5 MJ/kg during the starter phase, respectively. Although diet 5 (ME 15.5 MJ/kg) showed slightly lower weight gain compared to the other diets (13.5, 14.0, 14.5, 15.0 MJ/kg), it did not significantly affect overall growth performance. Brand et al. (2000) also conducted a trial that revealed diet composition had no significant effect on growth production. Significant difference was observed in the pre-starter phase, in which again, group treated with ME 15.5 MJ/kg (diet 5) showed less weight gain as compared to the other four groups. Overall, ostriches fed with diet 3 (ME 14.5MJ/kg) showed more weight gain. However, they observed increased feed intake when ostriches were offered a low-energy diet (9.0 MJ/kg), though feed conversion efficiency decreased between 4–11 months of age. Swart (1986) demonstrated that growing ostriches require 0.76 MJ of metabolisable energy (ME) to maintain body growth. In another study, Brand et al. (2014) reported better feed conversion ratios (FCRs) and improved growth performance when ostriches were fed medium-energy diets (7.0 MJ ME) during the pre-starter, starter, and growing phases. Improved growth and skin size were observed during the finishing phase with high-energy diets (11.5 MJ ME/kg) (Brand et al., 2014).

Nikravesh-Masouleh et al. (2018) also investigated the effects of different energy levels on various body parameters in ostriches. During the second week of their study, the highest breast, abdomen, and arm circumferences were recorded in chickens fed a diet containing 10 MJ/kg of energy, compared to those fed a 10.88 MJ/kg energy diet. It was a long-term effect because the observations remained similar during the study (week 2-9). The only deviation was in week 5 when body length was more in group fed with 2600 MJ/kg. Brand et al. (2003) offered three diets with different energy levels 8.5, 9.5, and 10.5 MJ ME/kg—during the breeding season and found that ostriches fed the lowest-energy diet gained only 4 kg, while those fed the highest-energy diets (9.5 and 10.5 MJ ME/kg) gained more weight. In another study, Nikravesh-Masouleh et al. (2018) offered two energy levels (2400 kcal/kg and 2600 kcal/kg) combined with three protein levels (20%, 24%, and 26%) to evaluate their effects on 12 body measurements from 2–9 weeks of age in 36 ostriches. The study revealed that ostriches fed a lower energy diet (2400 kcal/kg) had the highest body weight, breast length, and abdominal circumference during the 2–9 week period. These findings imply the importance of balancing dietary energy levels for optimal growth performance in ostriches, especially during different stages of growth. While lower-energy diets can increase feed intake, they may reduce overall growth efficiency, whereas higher-energy diets improve growth and body condition, particularly in the finishing phase.

### Influence of proteins on body characteristics and growth performance

Nutrition plays a critical role in the ostrich production system, with protein being a key component (Carstens et al., 2014). Protein is, however, an expensive ingredient in feed manufacturing, necessitating the use of alternative, low-cost feed sources that do not compete with human food supplies. Soybean meal is a commonly used protein source in ostrich diets, as it provides a balanced mix of essential amino acids, particularly arginine and leucine (Cooper et al., 2004; Abbas et al., 2023). An alternative to soybean as the primary protein source for ostriches or even poultry industry is expected soon (Tallentire et al., 2018). Brand et al. (2020a) found no decline in farming efficiency or ostrich performance when canola meal replaced soybean meal at 0%, 25%, 50%, 75%, and 100% levels. The study reported overall weights of 102 kg and 99 kg for ostriches fed 100% soybean meal and 100% canola meal, respectively, from day 77 to 337. Lysine and Methionine are often limited in feed ingredients, therefore are essential in poultry diets. Additionally, soybean meal has been shown to reduce the loss of mineral ions in poultry diets (Hirabayashi et al., 1998; Asghar et al., 2024). Table 4 shows effects of different protein levels on growth performance of ostriches. Ostrich chicks are typically fed diets containing 13–17% protein between the ages of 4 and 11 months, but research indicates that these differences in protein levels have no significant effect on their growth performance (Brand et al., 2000). Cilliers et al. (1997) observed the true digestibility of amino acids in ostriches to range from 0.780 to 0.862, which is slightly higher than the digestibility observed in cockerels (0.723–0.825) that was measured in a comparative study by Cilliers et al. (1997). Miao et al. (2003) reported net efficiency of different amino acids from 0.615 to 0.968 in ostriches. Glatz et al. (2008) compared the effects of low-energy (10.0 MJ/kg) and low-protein (126 g/kg) diets with high-energy (12.5 MJ/kg) and high-protein (143 g/kg) diets on ostrich growth performance. The results showed that ostriches fed the lower-energy and protein diet gained more weight (277.3 g/day) compared to those groups fed the higher-energy and protein diet (max 50.9g/bird). Protein requirements decrease as ostriches age and gain weight. For example, Gous and Brand (2008) recorded protein requirements of 14.71% for growing ostriches and 12.15% for finishing birds. Research also shows that feed conversion is more efficient when ostriches are given diets containing 22.5% or 17.5% CP at 10–14 weeks of age, with these birds consuming more feed compared to those on diets containing 12.5% CP (Ahmed et al., 2012). Table 4 illustrates the impact of increasing dietary protein levels on tibiotarsus girth, with the highest value recorded at 18% CP (18.38 cm) and a protein efficiency ratio (PER) of 1.63%, compared to 1.23% for 21% CP and 1.01% for 24% CP. The suggested levels of energy and protein for starter diets are 13.5MJ/Kg and 22%, respectively, to achieve optimal growth and production in ostriches. Scheideler (1994) reported that CP levels between 15% and 24% are ideal for young ostriches, with protein requirements decreasing as the birds mature. Ahmed et al. (2012) found that ostrich chicks fed diets with 17.5% or 22.5% CP consumed more feed and had better FCR compared to birds on a 12.5% CP diet. However, the differences between the 17.5% and 22.5% CP diets were not significant in terms of feed consumption or FCR. A similar CP level of 180-210 g/kg is also proposed for optimal ostrich growth (Mahrose et al., 2019). However, higher protein treatments (22% and 24%) were found to increase serum albumin and globulin levels in ostriches (Nikravesh-Masouleh, et al., 2021). This study did not reveal if the values of albumin and globin were beyond the normal range. Moreover, the effect of high total protein (albumin/globulin) levels in feed has been investigated in chicken and quails (Flora et al., 2000; Alabdallah et al., 2021), but no such investigations were found in the literature for ostriches.

**Table 4:** Effects of proteins on body characteristics and growth performance.

Performance objectives	Protein Levels tested^1^	Optimal level	Age	Sex	Effects	References
BWG (kg)	18, 21, 24	18	2-9 weeks	Unsexed	+19.6	(Mahrose et al., 2015)
	132, 160, 175	175	147 days	Straight Run	ND^2^	(Brand, 2012)
	13.5, 15, 16.5	13.5	Adult	Male	+1.78	(Brand et al., 2002)
Thoracic Girth (cm)	13.5, 15, 16.5	13.5	Adult	Male	+1.50	(Brand et al., 2002)
Feed Conversion Ratio	14, 16, 18	14	Adult	Straight Run	+9.90	(Swart and Kemm, 1985)
Abdomen Circumference	20, 22, 24	22	9 weeks	Straight Run	+4.50	(Nikravesh-Masouleh et al., 2018)
Thigh Circumference	20, 22, 24	22	9 weeks	Straight Run	+1.20	(Nikravesh-Masouleh et al., 2018)
Tibiotarsus Girth (cm)	18, 21, 24	18	2-9 weeks	Unsexed	+48.2	(Mahrose et al., 2015)

1 = g/kg, 2 = Not determined

Mahrose et al. (2015) studied the effects of different protein levels on tibiotarsus girth and length in ostriches, concluding that increasing protein levels reduced tibiotarsus girth, while tibiotarsus length was not significantly affected by dietary protein levels between 2 and 9 weeks of age. In another study, Mahrose et al. (2019) reported that increased crude protein levels can reduce tibiotarsus length. Swart and Kemm (1985) conducted a study on growing ostriches weighing between 60 and 110 kg, providing diets with varying protein levels (14%, 16%, and 18%) and lucerne content (67%, 57%, and 35%). The best overall performance, with a 235 g/day weight gain and a 10:1 FCR, was achieved with a diet containing 14% protein and 35% lucerne, which had a ME value of 10.8 MJ ME/kg. Mahrose et al. (2015) also reported the highest FCR (1.63%) in ostriches consuming an 18% protein diet, followed by 1.23% for a 21% protein diet and 1.01% for a 24% protein diet from 2–9 weeks of age. Bovera et al. (2011) demonstrated that ostriches require low to medium levels of protein (16–18%) for effective growth. While protein is essential for ostrich growth, optimal levels vary with age and growth stages. Feeding higher protein levels may not always result in improved growth or feed efficiency (Brand et al., 2020b), and care must be taken to avoid excessive protein intake to minimize environmental impacts.

### Influence of fiber on growth performance

Ostrich birds have a unique ability to digest plant fiber more efficiently than other poultry species due to their long large intestine, especially the cecum (Hastings and Farrell, 1991). Growing ostriches are capable of converting up to 76% of fibrous content into metabolizable energy (Cooper et al., 2005). Crude fiber is one of the most important ingredients in ostrich feed, and the optimal fiber content varies by age, ranging from 6% to 18% (Cooper et al., 2005). Jensen et al. 1992 recommend an overall <10% fiber content in the ostrich diet. Finishing ration may contain roughage up to 70% and pre-slaughter ration up to 90% (Cooper et al. 2005). Angel et al. 1994 reported a linear increase in digestibility of neutral detergent fiber (NDF) in ostriches with 33% inclusion from 3 weeks to 30 months of age. Young ostriches (3 months) may suffer from impaction if fed high levels of fiber (Cooper et al., 1999b). So, the fiber source should be from high quality source. The hindgut of ostriches houses microflora that help in the digestion of plant fibers, primarily hemicellulose (66%) and cellulose (38%) (Cilliers, 1998). Lucerne hay is a common roughage source in ostrich diets, providing 18% CP, 30% crude fiber, and 8.9 MJ/kg of apparent metabolizable energy (AME). The total metabolizable energy for lucerne is 8.9 MJ/kg (Cilliers et al., 1998). Cilliers et al. 2010 also reported apparent metabolizable energy of lucerne 8.9 MJ/kg for ostriches. Swart (1988) conducted a trial that determined the digestibility coefficients for cellulose (0.38%) and hemicellulose (0.66%) when ostriches were offered 340 g of Lucerne per kilogram of feed. Some researchers have recommended using wheat bran as a good substitute for Lucerne bran in ostrich diets with ME 11.9 MJ/kg on dry basis (>8.9 MJ/kg by lucerne) (Miao et al., 2003; Cilliers, 1994). The end products of fiber fermentation provide about 76% of ME required for ostriches in the growing stage (Swart et al., 1993a). Fiber fermentation in ostriches begins to develop properly around 10 weeks of age, with younger birds showing limited ability to digest fiber (Matsui et al., 2010). Compared to poultry, ostriches are more efficient in utilizing feed from high-fiber diets. In ostriches, food moves slowly through the hindgut (caeca and colon) because of the considerable length of the hindgut (2/3) of the overall GIT, allowing microbial feed contents more exposure for fermentation. By 30 days of age, 57% of the entire large intestine is involved in fiber digestion. However, organ maturation may respond differently to various diets, as reported by (Abd El-Wahab et al., 2021). The study found that ostriches at 15 months of age, fed corn silage and pelleted feed, had higher weights of empty gizzard and empty caecum compared to those fed haylage and pelleted feed.

As herbivores, ostriches rely heavily on their hindgut to retain dietary fiber and provide the right environment (pH 6.9–7.3) for fermentation (Swart et al., 1993b). Their digestive system shares many similarities with herbivorous mammals, particularly in terms of gut capacity, feed intake, digesta passage time, digestibility of feed, and reduction in feed particle size (Fritz et al., 2011; Frei et al., 2015). Glatz et al. (2003) examined the effects of feeding ostrich’s pea straw-based diets, both with and without Lucerne supplementation. Ostriches fed a diet containing 24% pea straw and 20% Lucerne gained 54.3 kg, while those on a diet without Lucerne gained 57.7 kg from 24 to 30 weeks of age. When offered diets with 24% and 40% pea straw, birds achieved 50.1 kg and 48.9 kg of weight gain, respectively, by 27 weeks of age. Afshin et al. (2024) replaced alfalfa with *Berberis vulgaris* leaves and found no significant decrease in body characteristics and growth. However, a 50% replacement of barberry leaves with alfalfa remained favorable in terms of daily weight gain and feed conversion ratio (FCR) up to 150 days of growth in ostriches. This better FCR with 50% substitution might be because of the combination of different feeds with different digestion rates, which can enhance digestibility of overall feed (Soltan et al., 2021). Moreover, *Berberis vulgaris* leaves may produce secondary plant metabolites that alter nutrient utilization by the body (Afshin et al., 2024). Varastegani and Dahlan (2014) also demonstrated that feed intake and growth performance were positively influenced by the high inclusion of dietary fiber in ostrich feed, contributing to improved bird health and growth performance, due to positive impact of dietary fiber on gut microbiota modulation.

## Future implications

The ostrich industry has significant growth potential, particularly as the global demand for sustainable and alternative meat sources continues to rise. To fully realize this potential, it is essential to optimize the nutritional requirements of ostriches and implement effective feeding management strategies (Najafi et al., 2021). Based on the literature review, appropriate feed compositions should be used in ostrich farming. Being capable of digesting high fiber content, cost-effective feed ingredients may be added at high inclusion levels to meet energy and protein requirements. Through this study, protein content of more than 22% and energy level of more than 13.5 MJ/kg are not recommended practically. Future research should focus on precisely defining the dietary needs of ostriches across various developmental stages, including growth, breeding, and egg-laying periods (Jiang et al., 2020). Such efforts would not only enhance feed efficiency but also promote the overall health and productivity of ostriches. Feeding costs can be significantly reduced without compromising nutritional value through innovative feed formulations using locally available and cost-effective ingredients. Insect-based protein sources, such as black soldier fly larvae, are one promising alternative (Sajid et al., 2023). A breakthrough in the feasibility evaluation of insect-based feed for ostrich is required, as Abro et al. 2020 have initiated for the poultry industry in Kenya. Additionally, the development of advanced feeding technologies could facilitate personalized feeding programs for ostriches, ensuring that each bird receives the necessary nutrients. This would reduce waste and improve growth rates (Mirbehbahani et al., 2020).

Further research is needed to explore how various feeding strategies impact the quality of ostrich products, such as meat, leather, and other byproducts. Over time, animal feed technology is advancing for sustainable and cost-effective ingredients. It may cause a shift in basic feed components, including energy, protein, and fibrous sources (Van der Poel et al., 2020). Accurate, on-spot and fast feed testing can reshape the traditional laboratory analysis and lead to faster innovations (Van der Poel et al., 2020). Automated feeding systems (AFS) are being employed in poultry and ruminant farming (Romano et al., 2023). As ostrich farming continues to grow and contribute to meet requirements, research direction will shift towards AFS and precision in ostrich farming. A deeper understanding of nutrition's influence on ostrich products would enable farmers to adjust feeding practices to meet market demands more effectively. Embracing eco-friendly and organic feed ingredients, along with sustainable feeding practices, will position the ostrich industry as a leader in sustainable livestock production. Ostrich farming in an ecological extensive environment (pasture) will enhance its consumer acceptability and prove economic (Kistner 2019). However, it will require establishing quality pastures with rich fibrous content, and this strategy has the potential to raise sustainability globally. By focusing on these areas, future research and innovations can contribute to the long-term sustainability and success of ostrich farming worldwide, ensuring its prosperity in the global agricultural landscape.

## Conclusions

The ostrich industry, still emerging in Australia, Africa, and certain Asian countries, holds significant potential, particularly given the high market value of its products such as meat, hides, and oil. To fully realize this potential, tailored diets and feeding strategies are essential, leveraging local feed resources and adapting to various production systems like feedlots and grazing. Fiber fiber-digesting feature of ostriches should be optimally utilized in feed formulations, and extensive (grazing) farming practices should be encouraged with quality fibrous components like lucerne. Optimal feeding strategies should address current challenges, such as the efficient use of protein and energy levels, to enhance growth and minimize environmental impact. Evidence suggests that starting from 20% to 17% CP is adequate for ostrich chicks at different age groups, while excess protein contributes to nitrogen waste, necessitating balanced diets to prevent environmental pollution. Additionally, varying energy levels influence weight management during the breeding season, with high-energy diets promoting weight gain, whereas moderate energy diets support weight maintenance. Further research is essential to fine-tune nutrient requirements and feeding practices, ensuring sustainable and efficient ostrich farming. Individual effects of different vitamins and minerals on ostrich growth and reproduction need to be focused in the research area. Insect-based protein should be considered in the ostrich diet. Emphasizing eco-friendly feed components and advancing feeding technologies will pave the way for the industry's growth and sustainability, positioning ostrich farming as a leader in sustainable livestock production.

## Ethics approval

Not applicable.

## Consent for publication

Not applicable.

## Financial support statement

The present study was supported by the following project: “Innovation, digitalisation and sustainability for the diffused economy in central Italy”- code ECS00000041 - VITALITY (CUP C43C22000380007).

## Disclosures

The authors declare that they have no known competing financial interests or personal relationships that could have appeared to influence the work reported in this paper.

## References

[bib0015] Abbas G., Abbas S.W. (2018). Health and hygiene guidelines for ostriches. Int. J. Anim. Husb. Vet. Sci..

[bib0018] Abbas G., Mahmood S., Sajid M., Ali Y. (2017). Ostrich farming: a new turn in poultry industry of Pakistan. Adv. Zool. Bot..

[bib0001] Abbas G., Zahid O., Khan M., Sajid M., Asif M., Saeed H. (2018). Future of ostrich farming in Pakistan. Adv. Zool. Bot..

[bib0016] Abbas G., Ur Rehman Qureshi C.M., Asif M., Sajid M., Abbas S.W., Zahid O., Saeed H. (2018). Ostrich industry: a beautiful U-turn in poultry industry of Pakistan. Int. J. Anim. Husb. Vet. Sci..

[bib0017] Abbas G., Imran M.S., Ali M., Arshad M., Jabbar M.A., Mustafa A., Sultan Z., Hussain I., Shafique A., Laghari M.I., Niazi Z.K., Hussain M., Ali N., Mohyuddin S.G., Jaffery S., Al-Taey D.K.A., Ali H. (2023). Effect of soybean unavailability situations and COVID-19 on the poultry industry of Pakistan: a comprehensive analysis problems faced and its solution for sustainable animal production. Pak. J. Sci..

[bib0002] Abd El-Wahab A., Schuchmann F.F., Chuppava B., Visscher C., Pfarrer C., Kamphues J. (2021). Studies on the weight of the gastrointestinal tract, digesta composition and occurrence of gastro-and enteroliths in adult domesticated ostriches fed different diets. Poult. Sci..

[bib0003] Afshin M., Afzali N., Hosseini-Vashan S.J., Hajibabaei A., Ghavipanje N., Vargas-Bello-Peréz E. (2024). Implication of dietary barberry (Berberis Vulgaris) leaves inclusion on growth performance, nutrient digestibility, and carcass traits in ostriches. Sci. Rep..

[bib0019] Aganga A.A., Aganga A.O., Omphile U.J. (2003). Ostrich feeding and nutrition. Pak. J. Nutr..

[bib0005] Ahmed F.M., Salih R.M., Mohamed A. (2012). Some behavioral traits of red neck ostrich under captive conditions. World’s Vet. J..

[bib0020] Akram M.B., Khan M.I., Khalid S., Shoaib M., Azeema S. (2019). Quality and sensory comparison of ostrich and goat meat. Int. J. Life Sci..

[bib0006] Akram M., Asghar M., Jalal H. (2021). Essential oils as alternatives to chemical feed additives for maximizing livestock production. J. Hellen. Vet. Med. Soc..

[bib0009] Al-Khalifa H., Al-Naser A. (2014). Ostrich meat: production, quality parameters, and nutritional comparison to other types of meats. J. Appl. Poultry Res..

[bib0008] Alagawany M., Ashour E.A., El-Kholy M.S., Abou-Kassem D.E., Roshdy T., Abd El-Hack M.E. (2022). Consequences of varying dietary crude protein and metabolizable energy levels on growth performance, carcass characteristics and biochemical parameters of growing geese. Anim. Biotechnol..

[bib0021] Angel C.R. (1993). Proc. Assoc. Avian Vet., January 1993.

[bib0022] Arain M.A., Hassan F.U., Ozdemir F.A., Khaskheli G.B., Malik Z., Buzdar J.A., Fazlani S.A. (2025). Ostrich meat: a review on nutritional properties and health benefits. Food Sci. Anim. Resour..

[bib0011] Aryal B., Khanal L. (2022). Hatching success and chick mortality of the ostrich (Struthio camelus) in a commercial farm in Nepal. Sri Lankan J. Biol..

[bib0023] Asadi S., Ghaffari M.H., Mohammadipour S., Ghorbanpour A., Jafari A. (2024). Antibiotic resistance pattern of Escherichia coli NM O157 strains isolated from ostrich feces in Lorestan Province. Ostrich.

[bib0013] Asghar M.U., Rahman A., Hayat Z., Rafique M.K., Badar I.H., Yar M.K., Ijaz M. (2021). Exploration of *Zingiber officinale* effects on growth performance, immunity and gut morphology in broilers. Braz. J. Biol..

[bib0012] Asghar M.U., Doğan S.C., Wilk M., Korczyński M. (2022). Effect of dietary supplementation of black cumin seeds (Nigella sativa) on performance, carcass traits, and meat quality of Japanese quails (Coturnix coturnix japonica). Animals.

[bib0014] Asghar M.U., Sajid Q.U.A., Wilk M., Konkol D., Korczyński M. (2024). Sciendo. Ann. Anim. Sci..

[bib0024] Bels V. (2006).

[bib0025] Bezuidenhout, A. J., W. Burger, J. F. Putterill, and M.-L. Penrith. 1994. Evidence for cryptosporidial infection as a cause of prolapse of the phallus and cloaca in ostrich chicks (Struthio camelus). PMID: 12345678. 7501359

[bib0027] Bo H.X., Hanh H.Q., Hue D.T., Hoa D.V., Hoa N.T., Cong N.T., Luc D.D. (2025). Modeling growth curves to estimate suitable slaughter age for ostriches (Struthio camelus). Trop. Anim. Health Prod..

[bib0026] Bovera F., Marono S., Di Meo C., Iannaccone F., Attia Y.A., Nizza A. (2011). Comparison of caecal and faeces fermentation characteristics of ostrich by in vitro gas production technique. Acta Agric. Scand., Section A — Anim. Sci..

[bib0028] Bovera F., Nizza S., Attia Y.A., Di Meo C., Piccolo G., Nizza A. (2014). Prediction of digestible energy and gross energy digestibility of feeds and diets in ostriches. Br. Poult. Sci..

[bib0034] Brand T., Olivier A. (2011).

[bib0037] Brand T.S., Steyn H.H. (2002). Electronic feeding system for ostriches. South Afr. Pat..

[bib0033] Brand T.S., Nell C.J., Van Schalkwyk S.J. (2000). The effect of dietary energy and protein level on the production of growing ostriches. S. Afr. J. Anim. Sci..

[bib0029] Brand Z., Brand T.S., Brown C.R. (2003). The effect of dietary energy and protein levels on production in breeding female ostriches. Br. Poult. Sci..

[bib0035] Brand T.S., Olivier T.R., Gous R.M. (2012). The response in food intake and reproductive parameters of breeding ostriches to increasing dietary energy. S. Afr. J. Anim. Sci..

[bib0030] Brand T.S., Carstens P.D., Hoffman L.C. (2014). The effect of dietary energy concentrations on production variables of ostrich chicks (*Struthio camelus* var. *domesticus*). Br. Poult. Sci..

[bib0036] Brand T.S., Olivier T.R., Gous R.M. (2015). The reproductive response of female ostriches to dietary protein. Br. Poult. Sci..

[bib0039] Brand T.S., Viviers S.F., van der Merwe J., Hoffman L.C. (2018). The influence of different dietary energy concentrations on the production parameters of feedlot ostriches. S. Afr. J. Anim. Sci..

[bib0040] Brand T.S., Viviers S.F., Van der Merwe J., Hoffman L.C. (2019). Effect of varying levels of protein concentration on production traits of ostriches (Struthio camelus var. domesticus). S. Afr. J. Anim. Sci..

[bib0031] Brand T.S., van Der Merwe J., Hoffman L.C. (2020). Effect of including canola meal in diets of slaughter ostriches (Struthio camelus var. domesticus). S. Afr. J. Anim. Sci..

[bib0032] Brand T.S., Kritzinger W.J., Jordaan L., Hoffman L.C. (2020). The effect of varying dietary nutrient densities on the relative growth of ostrich body components. J. S. Afr. Vet. Assoc..

[bib0038] Brand T.S., Van der Merwe D.A., Hoffman L.C., Van der Westhuyzen J.P., Kritzinger W.J., Muller A. (2020). The effect of dietary energy and protein level on feather, skin and nodule growth of the ostrich (Struthio camelus). J. S. Afr. Vet. Assoc..

[bib0041] Brassó D.L., Béri B., Komlósi I. (2020). Studies on ostrich (Struthio Camelus)-review. Acta Agraria Debreceniens..

[bib0042] Brassó L.D., Szabó V., Komlósi I., Pusztahelyi T., Várszegi Z. (2021). Preliminary study of slaughter value and meat characteristics of 18 months ostrich reared in hungary. Agriculture.

[bib0043] Brassó L.D., Török E., Komlósi I., Posta J. (2022). Factors affecting the survival of Ostrich from hatching untilthe age of 48 weeks. Agriculture.

[bib0044] Brown C., Brand Z., Brand T. (2002). The effect of dietary energy and protein levels on body condition and production of breeding male ostriches (Struthio cemelus domesticus). S. Afr. J. Anim. Sci..

[bib0045] Bubier N.E., Lambert M.S., Deeming D.C., Ayres L.L., Sibly R.M. (1996). Time budget and colour preferences (with specific reference to feeding) of ostrich (struthio camelus) chicks in captivity. Br. Poult. Sci..

[bib0046] Bunter K.L., Cloete S.W. (2004). Genetic parameters for egg-, chick-and live-weight traits recorded in farmed ostriches (Struthio camelus). Livest. Prod. Sci..

[bib0047] Carstens P.D., Sharifi A.R., Brand T.S., Hoffman L.C. (2014). The growth response of ostrich (Struthio camelus var. domesticus) chicks fed on diets with three different dietary protein and amino acid concentrations. Br. Poult. Sci..

[bib0048] Cecil H.C. (1981). Reproductive performance of male turkeys fed low protein diets during the breeder period. Poult. Sci..

[bib0052] Cilliers S.C., du Preez J.J., Maritz J.S., Hayes J.P. (1995). Growth curves of ostriches (Struthio camelus) from Oudtshoorn in South Africa. Anim. Sci..

[bib0051] Cilliers S.C., Hayes J.P., Chwalibog A., Du Preez J.J., Sales J. (1997). A comparative study between mature ostriches (Struthio camelus) and adult cockerels with respect to true and apparent metabolisable energy values for maize, barley, oats and triticale. Br. Poult. Sci..

[bib0049] Cilliers, S. C. 1994. Evaluation of feedstuffs and the metabolisable energy and amino acid requirements for maintenance and growth in ostriches (Struthio camelus).

[bib0050] Cilliers, S. C. 1998. Feedstuff evaluation, metabolisable energy and amino acid requirements for maintenance and growth in ostriches. 12-23.

[bib0056] Cooper R.G., Horbañczuk J.O. (2002). Anatomical and physiological characteristics of ostrich (Struthio camelus var. domesticus) meat determine its nutritional importance for man. Anim. Sci. J..

[bib0058] Cooper S.M., Palmer T. (1994). Observations on the dietary choice of free-ranging juvenile ostriches. Ostrich.

[bib0061] Cooper R.G., Horbanczuk J.O., Fujihara N. (2004). Nutrition and feed management in the ostrich (Struthio camelus var. domesticus). Anim. Sci. J..

[bib0060] Cooper R.G. (1999). Ostrich meat, an important product of the ostrich industry: a southern African perspective. World’s Poult. Sci. J..

[bib0054] Cooper R.G. (2000). Critical factors in ostrich (Struthio camelus australis) production: a focus on southern Africa. World’s Poultry Sci. J..

[bib0055] Cooper R.G. (2007). Global ostrich industry: current state and future possible developments. Anim. Prod. Anim. Sci. Worldwide.

[bib0062] Coyne J.M., Evans R.D., Berry D.P. (2019). Dressing percentage and live weight to carcass weight differential in cattle: genetic and non-genetic influences. J. Anim. Sci..

[bib0063] Dalle Zotte A., Brand T.S., Hoffman L.C., Schoon K., Cullere M., Swart R. (2013). Effect of cottonseed oilcake inclusion on ostrich growth performance and meat chemical composition. Meat. Sci..

[bib0066] Deeming D.C., Ayres L., Ayres F.J. (1993). Observations on the commercial production of ostrich (Struthio camelus) in the United Kingdom: rearing of chicks. Vet. Rec..

[bib0067] Degen A.A., Kam M., Rosenstrauch A. (1989). Time-activity budget of ostriches (*Struthio camelus*) offered concentrate feed and maintained in outdoor pens. Appl. Anim. Behav. Sci..

[bib0068] Degen A.A., Kam M., Rosenstrauch A., Plavnik I. (1991). Growth rate, total body water volume, dry-matter intake and water consumption of domesticated ostriches (Struthio camelus). Anim. Sci..

[bib0069] Dikmen B.Y. (2024). Effects of season, sex and time of day on ostrich breeder (Struthio camelus) BEHAVIOR. JAPS: J. Anim. Plant Sci..

[bib0070] Dryden G.M. (2008).

[bib0065] Du Preez J.H., Terblanche S.J., Giesecke W.H., Maree C., Welding M.C. (1991). Effect of heat stress on conception in a dairy herd model under South African conditions. Theriogenology.

[bib0144] du Preez, J. J.. 1991. Ostrich nutrition and management.

[bib0071] Edalatian Dovvom M.S., Ghaniei A., Mojaver M.Jamshidian, Tohidi E. (2024). Genotypic and phenotypic characteristics of the phylogenetic groups of Escherichia Coli isolates from ostriches in Iran. Iran. J. Vet. Sci. Technol..

[bib0073] Elobeid E.A., Faki A.E., Amin A.E. (2022). Behavioral patterns during the grower period of captive red-necked ostrich (Struthio camelus). J. Fac. Sci. Technol..

[bib0074] Elsayed M. (2009). Effect of month of production on external and internal ostrich egg quality, fertility and hatchability. Egypt. Poultry Sci. J..

[bib0075] Fair M., Van Wyk J., Cloete S. (2012). Pedigree analysis of an ostrich breeding flock. S. Afr. J. Anim. Sci..

[bib0076] Farooq M. (2024). Study of diagnosis, associated risk factors and in-vitro antibiotic susceptibility of Salmonella isolated from ostrich (Struthio camelus). Pak. J. Sci..

[bib0077] Farrell, D. J. 1997. Nutritional research into ostriches: what we need to know.Pages 6–10 in Australian Ostrich Convention.

[bib0078] Ferguson N.S. (2006).

[bib0079] Flora P., Sangeetha R. (2000). Influence of growth stages and gender on serum glucose, albumin, globulin and total proteins in RIR chicken. Indian J. Poult. Sci..

[bib0080] Frei S., Ortmann S., Reutlinger C., Kreuzer M., Hatt J.-M., Clauss M. (2015). Comparative digesta retention patterns in ratites. Auk: Ornithol. Adv..

[bib0081] Fritz J., Hummel J., Kienzle E., Wings O., Streich W.J., Clauss M. (2011). Gizzard vs. teeth, it’s a tie: food-processing efficiency in herbivorous birds and mammals and implications for dinosaur feeding strategies. Paleobiology.

[bib0082] Gandini G., Burroughs REJ &., Ebedes H. (1986). Preliminary investigation into the nutrition of ostrich chicks (Struthio camelus) under intensive conditions. J. S. Afr. Vet. Assoc..

[bib0084] Glatz P.C., Ru Y.J., Hastings M.Y., Black D., Rayner B. (2003). On farm assessment of high fibre dietary sources for grower and finisher ostriches. Int. J. Poult. Sci..

[bib0083] Glatz P.C., Miao Z.H., Rodda B.K., Wyatt S.C. (2008). Effect of diets with different energy and protein levels on performance of grower ostriches. Aust. J. Exp. Agric..

[bib0085] Gous R.M., Brand T.S. (2008). Simulation models used for determining food intake and growth of ostriches: an overview. Aust. J. Exp. Agric..

[bib0087] Hallam M.G. (1992).

[bib0086] Hastings M.Y., Farrell D.J. (1991). A history of ostrich farming–its potential in Australian agriculture. Recent Adv. Anim. Nutr. Austr..

[bib0088] Hayter D. (1998). Veterinary problems of ostriches. Farm. World.

[bib0089] Heitmann S. (2020).

[bib0090] Henry S.G., Abou-El-Hawa S.H., Ramadan B.R., Khalifa A.H. (2024). Nutritional quality of Ostrich meat, edible offal, and fat tissue. Assiut J. Agric. Sci..

[bib0091] Hirabayashi M., Matsui T., Yano H., Nakajima T. (1998). Fermentation of soybean meal with Aspergillus usamii reduces phosphorus excretion in chicks. Poult. Sci..

[bib0092] Hoffman L.C. (2012). Advances in the electrical stunning and bleeding of ostriches. Anim. Welfare.

[bib0096] Horbañczuk J., Sales J., Celeda T., Konecka A., Ziêba G., Kawka P. (1998). Cholesterol content and fatty acid composition of ostrich meat as influenced by subspecies. Meat. Sci..

[bib0094] Horbanczuk J.O., Cooper R.G. (2001). Nutritive value of ostrich meat. World Poultry.

[bib0095] Horbanczuk J., Sales J., Celeda T., Konecka A., Zieba G., Kawka P. (1998). Cholesterol content and fatty acid composition of ostrich meat as influenced by subspecies. Meat. Sci..

[bib0093] Horbanczuk J. (2002).

[bib0097] Iorgoni V., Iancu I., Popa I., Gligor A., Orghici G., Sicoe B., Dreghiciu C., Purec D., Nistor P., Florea B., Herman V. (2025). First report of respiratory infection caused by multidrug-resistant Escherichia coli in an ostrich in Romania. Antibiotics.

[bib0099] Ipek A., Șahan Ü. (2006). Egg production and incubation results of ostrich farms in the Marmara region of Turkey. Arch. Geflügelkunde.

[bib0100] Irfan M., Mukhtar N., Ahmad T., Munir M.T. (2020). Gastric impaction: an important health and welfare issue of growing ostriches. Agric. Trop. Subtrop..

[bib0101] Jalal H., Doğan S.C., Giammarco M., Cavallini D., Lanzoni L., Pezzi P., Akram M.Z., Fusaro I. (2024). Evaluation of dietary supplementation of garlic powder (*Allium sativum*) on the growth performance, carcass traits and meat quality of Japanese quails (*Coturnix coturnix japonica*). Poult. Sci..

[bib0102] Jensen J.M., Johnson J.H., Weiner S.T. (1992).

[bib0103] Jesch E.D., Carr T.P. (2017). Food ingredients that inhibit cholesterol absorption. Prev. Nutr. Food Sci..

[bib0106] Jiang G., Ameer K., Kim H., Lee E.-J., Ramachandraiah K., Hong G.-P. (2020). Strategies for sustainable substitution of livestock meat. Foods..

[bib0104] Jimenez-Moreno E., Frikha M., de Coca-Sinova A., García J., Mateos G. (2013). Oat hulls and sugar beet pulp in diets for broilers. 1. Effects on growth performance and nutrient digestibility. Anim. Feed. Sci. Technol..

[bib0105] Johnson A.L. (2015). Sturkie’s Avian Physiology.

[bib0107] Jones S.D.M., Robertson W.M., Brereton D. (1995). Agriculture and Agri-Food Canada.

[bib0110] Lambrechts H., Huchzermeyer F.W., Swart D. (1998). Proceedings of the Second International Scientific Ratite Congress.

[bib0111] Li M., Abouelfetouh M.M., Salah E., Kiani F.A., Nan S., Ding M., Ding Y. (2024). Chicory supplementation improves growth performance in juvenile ostriches potentially by attenuating enteritis. Front. Vet. Sci..

[bib0109] Lilic S., Milijasevic J.B., Geric T. (2021). Dressing percentage and meat yield of Hybro G+ provenance broilers. IOP Conf. Ser.: Earth Environ. Sci..

[bib0117] Mahrose K.M., Attia A.I., Ismail I.E., Abou-Kassem D.E., Abd El-Hack M.E. (2015). Growth performance and certain body measurements of ostrich chicks as affected by dietary protein levels during 2–9 weeks of age. Open. Vet. J..

[bib0118] Mahrose K.M., EL-HACK M.E.A., Amer S.A. (2019). Influences of dietary crude protein and stocking density on growth performance and body measurements of ostrich chicks. Ana. Acad. Bras. Ciênc..

[bib0119] Majewska D., Jakubowska M., Ligocki M., Tarasewicz Z., Szczerbińska D., Karamucki T., Sales J. (2009). Physicochemical characteristics, proximate analysis and mineral composition of ostrich meat as influenced by muscle. Food Chem..

[bib0120] Matsui H., Ban-Tokuda T., Wakita M. (2010). Detection of Fiber-digesting bacteria in the Ceca of Ostrich using specific primer sets. Curr. Microbiol..

[bib0121] Meawad S.M. (2020).

[bib0113] Medina, F. X., and E. Aguilar Moreno. 2014. Ostrich meat: nutritional, breeding, and consumption aspects. The case of Spain.

[bib0122] Miah A.G., Abdulle K.M., Rahman M., Salma U. (2020). Growth performance and survivability of ostrich chicks in Bangladesh. J. Vet. Res. Adv..

[bib0123] Miao Z., Glatz P., Ru Y. (2003). The nutrition requirements and foraging behaviour of ostriches. Asian-Australas. J. Anim. Sci..

[bib0124] Milton S.J., Dean W.R.J., Siegfried W.R. (1994). Food selection by Ostrich in Southern Africa. J. Wildl. Manag..

[bib0125] Mirbehbahani S.M., Hosseini-Vashan S.J., Mojtahedi M., Farhangfar S.H., Hosseini S.A. (2020). Soluble and insoluble fibers in ostrich nutrition: influences on growth performance and blood biochemical indices during different ages. Trop. Anim. Health Prod..

[bib0115] Mitchell R.D., Edwards H.M. (1996). Effects of phytase and 1,25-dihydroxycholecalciferol on phytate utilization and quantitative Ca and P requirements in young broilers. Poult. Sci..

[bib0126] Morris T.R., Gous R.M. (1988). Partitioning of the response to protein between egg number and egg weight. Br. Poult. Sci..

[bib0127] Morris C.A., Harris S.D., May S.G., Jackson T.C., Hale D.S., Miller R.K., Keeton J.T., Acuff G.R., Lucia L.M., Savell J.W. (1995). Ostrich slaughter and fabrication: 1. Slaughter yields of carcasses and effects of electrical stimulation on post-mortem pH1. Poult. Sci..

[bib0116] Mustafa F., Sajjad A., Tahir R., Ali M., Sajjad M., Abbasi A., Abd_Allah E.F. (2024). Use of Periplaneta americana as a soybean meal substitute: A step towards sustainable transformative poultry feeds. Insects..

[bib0128] Mutiga M.I., Muoria P.K., Kotut K., Karuri H.W. (2016). Behavioural patterns and responses to Human disturbances of wild Somali ostriches (Struthio molybdophanes) in Samburu, Kenya. Int. J. Adv. Res. (Indore).

[bib0129] Najafi S., Ghasemi H.A., Hajkhodadadi I., Khodaei-Motlagh M. (2021). Nutritional value of whole date waste and evaluating its application in ostrich diets. Animal..

[bib0130] Naser R.A.A., Al-Redah S.A.A., Ahmed E.S., Zghair F.S., Al-Ezzy A.I.A. (2024). Comparative anatomy, histology, histochemistry, and immunohistochemistry of the esophagus in ostrich (Struthio camelus) and turkey (Meleagris gallopavo). Adv. Anim. Vet. Sci..

[bib0131] Niemann G.J. (2018).

[bib0134] Nikravesh-Masouleh T., Seidavi A., Kawka M., Dadashbeiki M. (2018). The effect of dietary energy and protein levels on body weight, size, and microflora of ostrich chicks. Trop. Anim. Health Prod..

[bib0135] Nikravesh-Masouleh T., Seidavi A., Solka M., Dadashbeiki M. (2021). Using different levels of energy and protein and their effects on bodyweight and blood chemistry of ostriches. Vet. Res. Commun..

[bib0137] Okasha M.A., Attia A.I., Mahrose K.M. (2019). Ostrich breeding in China. Zagazig J. Agric. Res..

[bib0138] Olivier T.R., Brand T.S., Brand Z. (2009). Influence of dietary energy level on the production of breeding ostriches. S. Afr. J. Anim. Sci..

[bib0139] Olivier T.R., Brand T.S., Gous R.M. (2024). A review of the calculation of energy, protein and amino acid requirements for maintenance and egg production of breeding ostriches. World’s Poultry Sci. J..

[bib0140] Paxton C.G.M., Bubier N.E., Deeming D.C. (1997). Feeding and pecking behaviour in ostrich (Struthio camelus) chicks in captivity. Br. Poult. Sci..

[bib0142] Poławska, E., A. Półtorak, J. Wyrwisz, A. Wierzbicka, K. Gutkowska, J. Pomianowski, and S. D. Sme. 2014. Physical traits and fatty acid profile of ostrich meat enriched in n-3 fatty acids as influenced by refrigerated storage duration and packaging type.

[bib0141] Polat U., Cetin M., Turkyilmaz O., Ak I. (2003). The effects of different dietary protein levels on the biochemical and production parameters of ostriches (Struthio camelus). Veterinarski Arhiv.

[bib0143] Previti A., Sicuso D.A., Biondi V., Bsrat A., Pugliese M., Gebrekidan B., Passantino A. (2025). Developing an ostrich welfare assessment protocol (OWAP) in intensive and semi-intensive systems. Vet. Sci..

[bib0145] Prokopenko N., Melnyk V., Bazyvoliak S. (2021). Biological features of egg productivity of black African ostriches under a semi-intensive keeping. Ukrain. J. Ecol..

[bib0146] Ramedani Z., Alimohammadian L., Kheialipour K., Delpisheh P., Abbasi Z. (2019). Comparing energy state and environmental impacts in ostrich and chicken production systems. Environ. Sci. Pollut. Res..

[bib0147] Ramos S., Caetano S., Savegnago R.P., Nunes B., Ramos A.A., Munari D. (2013). Growth curves for ostriches (Struthio camelus) in a Brazilian population. Poult. Sci..

[bib0148] Romano E., Brambilla M., Cutini M., Giovinazzo S., Lazzari A., Calcante A., Tangorra F.M., Rossi P., Motta A., Bisaglia C., Bragaglio A. (2023). Increased cattle feeding precision from automatic feeding systems: considerations on technology spread and farm-level perceived advantages in Italy. Animals.

[bib0150] Sajid Q.U.A., Asghar M.U., Tariq H., Wilk M., Płatek A. (2023). Insect meal as an alternative to protein concentrates in poultry nutrition with future perspectives (an updated review. Agriculture.

[bib0151] Sales J., Sales J., Oliver-Lyons B. (1996). Ostrich Meat: a Review.

[bib0152] Sales J., Sales J., Oliver-Lyons B. (1996). Ostrich Meat: a Review.

[bib0153] Sales J. (1998). Fatty acid composition and cholesterol content of different ostrich muscles. Meat. Sci..

[bib0154] Salih M.E., Brand T.S., van Schalkwyk S., Blood J., Pfister B., Brand Z., Akbay R. (1998). Proceedings of the Second International Scientific Ratite Congress.

[bib0155] Scheideler S.E. (1994). Proceedings of the Annual Conference of the Association of Avian Veterinarians.

[bib0156] Schmitt J. (1997). Maximum growth of ostrich for slaughter. Austr. Ostrich Assoc. J..

[bib0164] Schou M.F., Engelbrecht A., Brand Z., Svensson E.I., Cloete S., Cornwallis C.K. (2022). Evolutionary trade-offs between heat and cold tolerance limit responses to fluctuating climates. Sci. Adv..

[bib0157] Shanawany M. (1996). Principles and practice of ostrich feeding. Feed Mix.

[bib0158] Siddique F., Abbas R.Z., Mahmood M.S., Iqbal A., Javaid A., Hussain I. (2020). Eco-epidemiology and pathogenesis of Newcastle disease in ostriches (Struthio camelus). World’s Poultry Sci. J..

[bib0165] Simela L., Webb E.C., Bosman M.J.C. (2011). Live animal and carcass characteristics of South African indigenous goats. S. Afr. J. Anim. Sci..

[bib0159] Smith, W. A. 1995. Feeding and feed management. Practical guide for ostrich management and ostrich products.

[bib0161] Swart D., Kemm E.H. (1985). Die invloed van dieetproteien-en energiepeil op die groeiprestasie en veerproduksie van slagvolstruise onder voerkraaltoestande. S. Afr. J. Anim. Sci..

[bib0162] Swart D., Mackie R.I., Hayes J.P. (1993). Fermentative digestion in the ostrich (Struthio camelus var. domesticus), a large avian species that utilizes cellulose1. S. Afr. J. Anim. Sci..

[bib0163] Swart D., Siebrits F.K., Hayes J.P. (1993). Growth, feed intake and body composition of ostriches (Struthio camelus) between 10 and 30 kg live mass 1. S. Afr. J. Anim. Sci..

[bib0160] Swart D. (1988).

[bib0168] Tallentire C.W., Mackenzie S.G., Kyriazakis I. (2018). Can novel ingredients replace soybeans and reduce the environmental burdens of European livestock systems in the future?. J. Clean. Prod..

[bib0167] Tasirnafas M., Seidavi A., Rasouli B., Kawka M. (2015). Effect of vegetable wastage and energy in ostrich chick diet on performance and hematology. Trop. Anim. Health Prod..

[bib0169] Tesselaar G.-M.A. (2015).

[bib0171] Tomuta R., Rada O., Fericean L.M. (2021). Ostrich reproduction behavior under farming conditions. Res. J. Agric. Sci..

[bib0170] Torkan A., Badouei M.A. (2024). Investigating the virulence-associated genes and antimicrobial resistance of Escherichia fergusonii isolated from diseased ostrich chicks. Comp. Immunol. Microbiol. Infect. Dis..

[bib0172] Ullrey D.E., Allen M.E. (1996). Nutrition and feeding of ostriches. Anim. Feed. Sci. Technol..

[bib0173] Van der Poel A.F.B., Abdollahi M.R., Cheng H., Colovic R., Den Hartog L.A., Miladinovic D., Hendriks W.H. (2020). Future directions of animal feed technology research to meet the challenges of a changing world. Anim. Feed. Sci. Technol..

[bib0174] Varastegani A., Dahlan I. (2014). Influence of dietary fiber levels on feed utilization and growth performance in poultry. J. Anim. Prod. Adv..

[bib0175] Videvall, E., S. J. Song, H. M. Bensch, M. Strandh, A. Engelbrecht, N. Serfontein, O. Hellgren, A. Olivier, S. Cloete, and R. Knight. 2019. Early-life gut dysbiosis linked to mass mortality in ostriches. bioRxiv:841742. 10.1186/s40168-020-00925-7 PMC7552511 33046114

[bib0176] Videvall E., Song S.J., Bensch H.M., Strandh M., Engelbrecht A., Serfontein N., Hellgren O., Olivier A., Cloete S., Knight R. (2020). Early-life gut dysbiosis linked to juvenile mortality in ostriches. Microbiome.

[bib0178] WHO and FAO (2003). Diet, nutrition and the prevention of chronic diseases. World Health Organ. Tech. Rep. Ser..

[bib0177] Williams J.B., Siegfried W.R., Milton S.J., Adams N.J., Dean W., du Plessis M.A., Jackson S. (1993). Field metabolism, water requirements, and foraging behavior of wild ostriches in the Namib. Ecology..

